# The Secretion of *Streptomyces monbaraensis* Transglutaminase From *Lactococcus lactis* and Immobilization on Porous Magnetic Nanoparticles

**DOI:** 10.3389/fmicb.2019.01675

**Published:** 2019-08-06

**Authors:** Tiange Ma, Jiaojiao Lu, Jing Zhu, Xingjiang Li, Hongwei Gu, Manuel Montalbán-López, Xuefeng Wu, Shuizhong Luo, Yanyan Zhao, Shaotong Jiang, Zhi Zheng, Dongdong Mu

**Affiliations:** ^1^School of Food and Biological Engineering, Key Laboratory for Agricultural Products Processing of Anhui Province, Hefei University of Technology, Hefei, China; ^2^State Key Laboratory of Tea Plant Biology and Utilization, School of Science, Anhui Agricultural University, Hefei, China; ^3^College of Chemistry, Soochow University, Suzhou, China; ^4^Department of Microbiology, Faculty of Sciences, University of Granada, Granada, Spain; ^5^Key Laboratory of Molecular Microbiology and Technology, Ministry of Education, College of Life Sciences, Nankai University, Tianjin, China

**Keywords:** transglutaminase, *Lactococcus lactis*, secretion, signal peptide SP*_usp45_*, immobilized enzyme

## Abstract

Microbial transglutaminase (MTG) from *Streptomyces mobaraensis* is an important enzyme widely applied in food processing for the improvement of protein properties by catalyzing the cross-linking of proteins. In this work we aimed at improving the production and enabling an easy and efficient purification process from culture supernatants. Thus, recombinant vectors, with either a constitutive promoter (P*_p5_*) or an inducible promoter (P*_nisA_*), controlling the expression of the MTG gene fused to the signal peptide of Usp45 (SP*_usp45_*) were constructed and then expressed in *Lactococcus lactis*. After purification, 43.5 ± 0.4 mg/L mature MTG-6His was obtained. It displayed 27.6 ± 0.5 U/mg enzymatic activity cross-linking soy protein isolate effectively. The purified mature MTG was immobilized with magnetic porous Fe_3_O_4_ nanoparticles, which improved its activity up to 29.1 ± 0.4 U/mg. The immobilized MTG maintained 67.2% of the initial activity after being recycled for 10 times. The high production and secretion of functional *S. mobaraensis* MTG from *L. lactis* and the magnetic immobilized MTG-6His onto Fe_3_O_4_ nanoparticles reported in this study would have potential industrial applications.

## Introduction

Transglutaminases are a kind of enzymes which catalyze the formation of an isopeptide bond between a γ-carboxyamide group of a glutamine residue and a primary amine, usually the 𝜀-amine of a lysine, intra/intermolecularly, by an acyl-transfer reaction (Gorman and Folk, 1981).

Transglutaminases are widespread distributed in organisms including mammals, plants, and microorganisms. Microbial transglutaminase (MTG) refers to transglutaminases produced by microorganism. The first MTG was discovered in *Streptomyces mobaraensis* where it is expressed as a zymogen consisting of a signal peptide, a 45 amino acids (Aa) pro-region and a 331 Aa mature peptide (Ando et al., 1989; Dickneite et al., 2015; Aloisi et al., 2016). Then it is translocated from the cytoplasm as an inactive pro-MTG, which is subsequently activated by proteolytic processing to release the mature transglutaminase (Pasternack et al., 1998). Because of its broad substrate specificity, relatively small molecular weight, and independence of Ca^2+^, MTG has been widely used as an industrial catalyst in many processes (Chen et al., 2013a,b). The major application of MTG is in the food industry to modify food proteins in order to enhance their physicochemical properties such as emulsification, water-holding capacity, viscosity, elasticity and foaming (Kieliszek and Misiewicz, 2014; Gaspar and de Goes-Favoni, 2015). Meanwhile, the application of MTG in dairy-based systems has increased to promote desired functional changes and to develop pleasing flavor in dairy products. For example, the formation of MTG-catalyzed crosslinking between milk proteins intra/intermolecularly leads to increased solubility, heat stability, gelation, and thickening and emulsifying activity thereby strengthening the functional properties of the product without any negative impacts on flavor and nutrition (Romeih and Walker, 2017).

Currently, MTG is mainly produced by conventional fermentation with *S. mobaraensis* as a host strain (Washizu et al., 1994). The use of the wild-type producer entails obstacles related to post-translational modification induced by self-produced active transglutaminase (Griffin et al., 2002) and complicated downstream procedure, resulting in an rather high price for the enzyme, which in turn hampers applications in food processing. Heterologous overexpression of genes of interest for functional studies and large quantities of MTG is worthy of attempting. As a model bacterium, *Escherichia coli* has been developed to be the most commonly used production system for heterologous proteins. However, the formation of inclusion bodies and failure to direct the secretion by SP*_PelB_* limited the MTG production in *E. coli* to a high-cost level (Yokoyama et al., 2000; Salis et al., 2015). Moreover, after application the residual MTG can not be recycled for reuse. Therefore, an effective secretion system to produce high-quality MTG and a convenient recycling method for MTG by immobilization are strongly desired.

**FIGURE 1:**
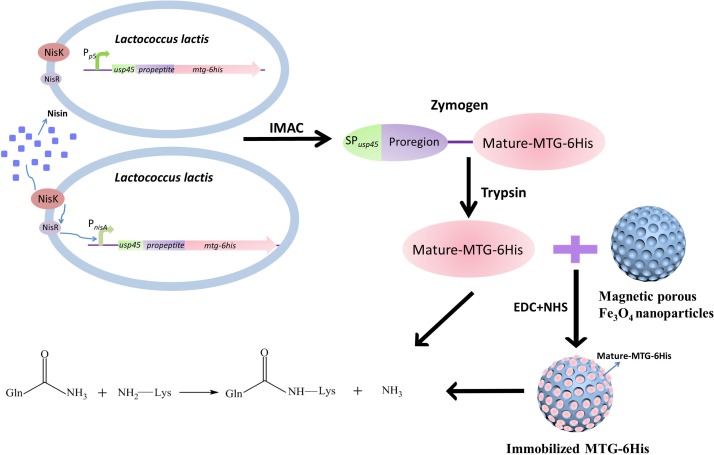
The basic experimental process.

*Lactococcus lactis* is a Gram-positive bacterium, which has been extensively applied in the production of buttermilk and cheese (Leroy and De Vuyst, 2004). As a host strain to produce heterologous proteins, *L. lactis* is receiving increasing interest in both food and clinical fields from biologists due to either its safe status [generally regarded as safe (GRAS)], or unique biological natures (possessing only one exported housekeeping protease, HtrA (Poquet et al., 2000), which would minimize the unexpected protein degradation). Given the fact that *L. lactis* secretes only one reported major extracellular protein, Usp45 (van Asseldonk et al., 1990), most heterologous proteins secreted by *L. lactis* are directed by SP*_usp45_* via the secretion (Sec) pathway (Nouaille et al., 2003). Magnetic mesoporous materials have been proved to be excellent carriers for immobilized enzyme for their outstanding magnetic property, high compatibility and modifiable surface. In this study, two MTG secretion systems were constructed by using the Sec pathway in *L. lactis* through SP*_usp45_* signaling: one involves the P*_p5_* constitutive promoter based on the plasmid pNZ8048-P*_p5_* (Zhu et al., 2015), and the other one involves the P*_nisA_* nisin-inducible promoter based on the plasmid pNZ8048 (de Ruyter et al., 1996). Additionally, the immobilization of the MTG by covalent binding to porous Fe_3_O_4_ has been performed, so as to improve the enzymatic activity of MTG and recover the MTG (Figure 1).

## Materials and Methods

### Materials

The information of the used strains and plasmids in this work are covered in Table 1. *S. mobaraensis* (CGMCC 4.5591, Beijing, China) was cultured in TSBY medium (Guan et al., 2015). *L. lactis* NZ9000 was cultured in M17 medium supplemented with 0.5% glucose (GM17). 1.5% agar was added when solid media were required. Chloramphenicol (5 μg/ml) was used when required for the culture of transformant *L. lactis* strains.

**Table 1:** Strains and vectors used in this work.

Strain or vector	Characteristic	Information	References
Strains *S. mobaraensis*	Used for amplification of *mtg* gene	MTG wild-type producer	(Ando et al., 1989) CGMCC 4.5591, (CGMCC, Beijing, China)
*L. lactis* NZ9000	Expression host strain	*pepN:nisRK*	Linares et al., 2010
Vectors			
pNZ8048	Vector including P*_nisA_*; Cm^R^	Inducible expression vector	de Ruyter et al., 1996
pNZ8048-*mature*-*mtg*	Recombinant vector; Cm^R^	Carries mature *mtg* gene from *S. mobaraensis*	This work
pNZ8048-*SP_usp45_(K2A)*-*promtg*	Recombinant expression vector; Cm^R^	Carries the gene of a mutated signal peptide SP*_usp45_* (K2A) fused to pro-MTG from *S. mobaraensis*	This work
pNZ8048-*SP_usp45_*-*promtg*	Recombinant expression vector; Cm^R^	Carries the gene of the signal peptide SP*_usp45_* fused to pro-MTG from *S. mobaraensis*	This work
pNZ8048-*SP_usp45_-promtg-mature-mtg*	Recombinant expression vector; Cm^R^	Carries the gene of the signal peptide SP*_usp45_* fused to pro-MTG from *S. mobaraensis* and mature MTG	This work
pNZ8048-P*_p5_*	Vector under control of P*_p5_*; Cm^R^	Promoter P*_nisA_* is replaced by Promoter P*_p5_*	Zhu et al., 2015
pNZ8048-P*_p5_*-*SP_usp45_(K2A)-promtg*	Recombinant expression vector; Cm^R^	Carries the gene of the mutated signal peptide SP*_usp45_* (K2A) fused to pro-MTG from *S. mobaraensis*	This work


*Cm^*R*^, chloramphenicol resistance.*

Iron chloride, sodium carbonate, sodium acetate trihydrate, ethylene glycol (EG), N-Hydroxysuccinimide (NHS) and N-(3-dimethylaminopropyl)-N-ethylcarbodiimide hydrochloride (EDC) were bought from Sigma-Aldrich.

### Molecular Manipulations

Molecular manipulations were carried out according to standard methods (Sambrook et al., 2001). *L. lactis* NZ9000 electrotransformation was applied based on methods established by Holo and Nes with a Gene Pulser^TM^ and Pulse Controller apparatus (Bio-Rad Laboratories, Hercules, CA, United States) (Holo and Nes, 1989). Restriction digestions and ligations were committed according to the instructions of manufacturer (Thermo Fischer).

### Construction of Recombinant Vectors Harboring *mtg*

**FIGURE 2:**
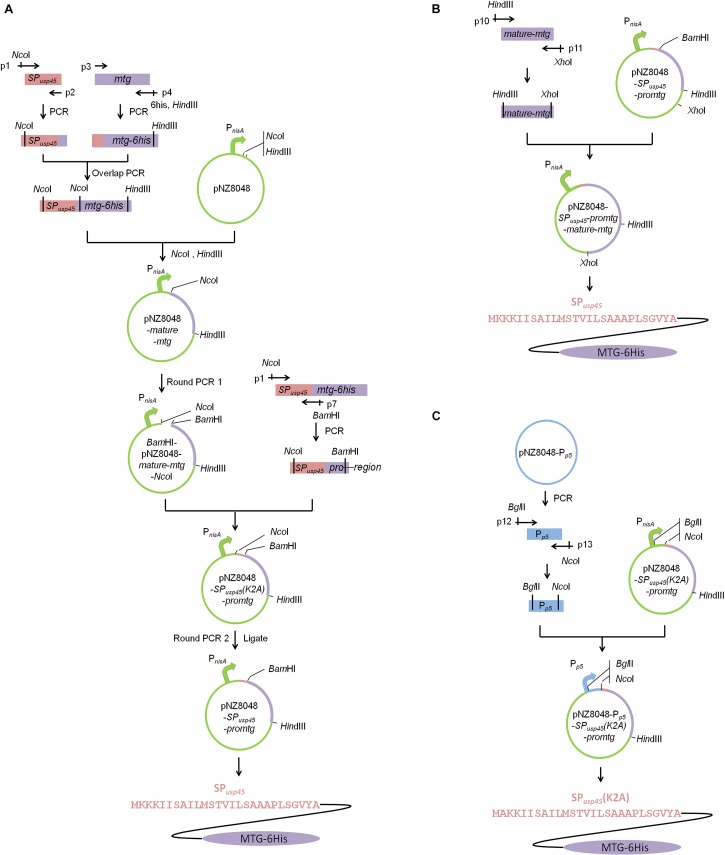
Schematic illustrations of vector constructions. Construction of pNZ8048-*SP_usp45_*-*promtg* and pNZ8048-P*_p5_*-*SP_usp45_(K2A)-promtg*. **(A)** The construction of pNZ8048-*SP_usp45_*-*promtg*, which promoter is P*_nisA_*. **(B)** The construction of pNZ8048-*SP_usp45_-promtg-mature-mtg*, which promoter is P*_nisA_*. **(C)** The construction of pNZ8048-P_p5_- *SP_usp45_(K2A)*-*promtg*, which promoter is P_p5_.

Extraction of genomic DNA was carried out using the genomic DNA purification kit (Takara). Plasmid isolation was performed with the plasmid DNA extraction kit (Takara). Primers used to amplify gene fragment are shown in Table 2. *S. mobaraensis* *mtg* gene (GenBank: accession number DQ132977) directed by the signal peptide of *L. lactis* SP*_usp45_* (GenBank: accession number ABY84357) and under the control of the nisin-inducible promoter was constructed as shown in Figure 2 by overlap PCR and round PCRs (Heckman and Pease, 2007). The genomic DNA of *L. lactis* NZ9000 (Linares et al., 2010) was used as the template to amplify the SP*_usp45_* gene fragment (*Nco*I*-*SP*_usp45_-overlapseq*) with primers p1 and p2. The genomic DNA of *S. mobaraensis* was used as the template to amplify the *mtg* fragment lacking its original signal peptide sequence and with a 6-His-tag (*mtg-6his-Hin*dIII) with primers p3 and p4. The gene encoding a hexahistidine tag fused upstream a *Hin*dIII site were added to the 5′ end of primer p4. In order to integrate these two gene fragments, primers p2 and p3 were synthesized reversely complementary so they overlap with each other (Niwa et al., 1996). Primers p1 and p4 were used to generate the fragment SP*_usp45_-mtg-6his* using as a template an equimolecular mixture of *Nco*I*-*SP*_usp45_-overlapseq* and *mtg-6his-Hin*dIII. After being digested by *Nco*I and *Hin*dIII, SP*_usp45_-proregion* was cleaved since there was a *Nco*I cleavage site between the *proregion* sequence and *mature-mtg*. *mature-mtg-6his* was inserted into pNZ8048, _ENREF_25and the resulting vector was designated as pNZ8048*-mature-mtg*. In order to correctly add the SP*_usp45_-proregion*, round PCR1 was used to introduce a *Bam*HI site to pNZ8048*-mature-mtg* to generate the fragment of *Bam*HI*-*pNZ8048*-mature-mtg-Nco*I with primers p5 and p6. The fragment SP*_usp45_-proregion* was amplified by primers p1 and p7 using SP*_usp45_-mtg-6his* amplicon as the template to generate the fragment of *Nco*I*-*SP*_usp45_-proregion-Bam*HI. Then the resulting two fragments *Bam*HI*-*pNZ8048*-mature-mtg-Nco*I and *Nco*I*-*SP*_usp45_-proregion-Bam*HI were digested using *Nco*I and *Bam*HI and ligated using T4 ligase as above (TransGen Biotech, Beijing, China), generating the expression vector pNZ8048-*SP_usp45_(K2A)*-*promtg*. The addition of the *Nco*I cleavage site in SP*_usp45_* caused a replacement of lysine 2 in the plasmid pNZ8048-*SP_usp45_(K2A)*-*promtg* into alanine, so the Round PCR2 was used to reestablish the wild-type sequence of SP*_usp45_*. Thus, the primers p8 and p9 were used for a round PCR2 and the product was ligated with T4 ligase to create the plasmid pNZ8048-*SP_usp45_*-*promtg* in which the recombinant pro-MTG-6His will be initiated by the nisin-inducible promoter P*_nisA_* (Figure 2).

**Table 2:** Primers used in this study.

Primer	Sequence (5’–3’)	Characteristic/function
p1	CATGCCATGGCAAAAAAGATTATCTCAGCTATTTTAA	*Nco*I cleavage site/for the amplification of *SP_usp45_*
p2	CTCTTCCCCCGCGCCATTGTCAGCGTAAACACCTGAC	Overlap the 5′ end of p3/for the amplification of *SP_usp45_*
p3	GTCAGGTGTTTACGCTGACAATGGCGCGGGGGAAGAG	Overlap the 5′ end of p2/for the amplification of *pro-mtg*
p4	CCCAAGCTTTCAGTGATGGTGATGGTGATGCGGCCAGCCCTGCTTTACCTTG	Codons of hexa histidine followed by a *Hin*dIII cleavage site/for the amplification of *pro-mtg*
p5	CGCGGATCCGACGACAGGGTCACCCCTCCCGC	*Bam*HI cleavage site/to insert cleavage site to pNZ8048*-mature-mtg*
p6	CCCTGTCCATGGTGAGTGCCTCC	*Nco*I cleavage site/to insert cleavage site to pNZ8048*-mature-mtg*
p1	CATGCCATGGCAAAAAAGATTATCTCAGCTATTTTAA	*Nco*I cleavage site/for the amplification of *SP_usp45_-pro-region*
p7	CGCGGATCCGGGGGCCCGGAACGACGG	*Bam*HI cleavage site/for the amplification of *SP_usp45_-pro-region*
p8	ATGAAAAAAAAGATTATCTCAGCTATTTTAATG	*5*′ *phosphorylation* and alanine was mutated to lysine/to reestablish the wild-type sequence of SP*_usp45_* on the plasmid pNZ8048-*SP_usp45_*-*promtg*
p9	GGTGAGTGCCTCCTTATAATTTATTTTG	To reestablish the wild-type sequence of SP*_usp45_* on the plasmid pNZ8048-*SP_usp45_*-*promtg*
p10	CCCAAGCTTCAAAATAAATTATAAGGAGGCAC	*Hin*dIII cleavage site/for the amplification of mature *mtg*
p11	CCGCTCGAGTCAGTGATGGTGATGGTGATGC	*Xho*I cleavage site/for the amplification of mature *mtg*
p12	GGAAGATCTGAAAAAGAAAATGTTTTTGTATTTTTAG	*Bgl*II cleavage site/for the amplification of *Promoter P_p5_*
p13	CATGCCATGGTGTAACCGTCCTCCTCAC	*Nco*I cleavage site/for the amplification of *Promoter P_p5_*


The promoter P*_p5_* was amplified by primers p10 and p11 using the plasmid pNZ8048-P*_p5_*_ENREF_24 (Zhu et al., 2015) as the template. A *Bgl*II site and a *Nco*I site were fused to the 5′ end of primer p10 and p11, respectively. Then, this amplicon and the plasmid pNZ8048-*SP_usp45_(K2A)*-*promtg* were digested by *Bgl*II and *Nco*I and P*_p5_* was cloned into pNZ8048-*SP_usp45_(K2A)*-*promtg* replacing P*_nisA_*. The resulting vector was designated as pNZ8048-P*_p5_*-*SP_usp45_(K2A)-promtg* in which transcription of the recombinant pro-MTG-6His will be initiated by the constitutive promoter P*_p5_* (Figure 2).

All the recombinant vectors were constructed in *L. lactis* NZ9000, extracted and checked by DNA sequencing.

### Growth Profile of *L. lactis*

*Lactococcus lactis* strains harboring each of the four vectors (pNZ8048-P*_p5_*, pNZ8048-P*_p5_*-*SP_usp45_(K2A)-promtg*, pNZ8048 and pNZ8048-*SP_usp45_*-*promtg*) or no vector were cultured in 6 mL of fresh GM17 medium with selective antibiotic or not at 30°C overnight. 100 μl of the seed culture was diluted with 50 mL of fresh GM17 and the growth profile was drawn according to the absorbance of the culture density at 600 nm every 2 h during 24 h. Fresh GM17 medium was used as blank control.

### Secretion of Pro-MTG-6His Under the Constitutive Promoter P*_p5_* in *L. lactis*

*Lactococcus lactis* NZ9000 harboring pNZ8048-P*_p5_*-*SP_usp45_(K2A)-promtg* was grown on GM17 agar plates containing 5 μg/ml (final concentration) chloramphenicol at 30°C overnight. A single colony was then picked and inoculated into 6 mL of fresh GM17 medium with selective antibiotic and cultured at 30 °C overnight. The seed culture of *L. lactis* NZ9000 harboring pNZ8048-P*_p5_*-*SP_usp45_(K2A)-promtg* was inoculated at a ratio of 1:50 into fresh fermentation medium (GM17) with selective antibiotic and cultured for 4, 12, 24, and 48 h. After centrifugation from the fermented samples at 9000 rpm for 20 min, the supernatant from various time intervals was obtained, just used for purified and then analyzed by reducing SDS-PAGE with Coomassie blue staining (Mu et al., 2018). Protein purification from 48 h fermented sample of *L. lactis* NZ9000 (pNZ8048-P*_p5_*) was treated as control.

### Secretion of Pro-MTG-6His Under the Inducible Promoter P*_nisA_* in *L. lactis*

An overnight culture of *L. lactis* NZ9000 harboring pNZ8048-*SP_usp45_*-*promtg* was inoculated at a ratio of 1:50 into fresh fermentation medium at 30°C. When the culture reached an OD of approximately 0.5 at 600 nm, the strains were induced by nisin with a final concentration of 1 ng/mL and grown for further 3, 12, 24, and 48 h. After centrifugation at 9000 rpm for 20 min, the supernatant from various time intervals was obtained, and then purified to be analyzed by SDS-PAGE (Mu et al., 2018). Protein purification from 48 h fermented sample of *L. lactis* NZ9000 (pNZ8048) was treated as control.

The concentration of P*_nisA_* inducer optimization for pro-MTG-6His expression in *L. lactis* was investigated as well. Overnight culture of *L. lactis* NZ9000 harboring pNZ8048-*SP_usp45_*-*promtg* was inoculated at a ratio of 1:50 into fresh fermentation GM17 medium in five parallel cultures. When the OD_600_ reached 0.5, each sample was induced at a final concentration of 1/3/5/7/9 ng/mL of nisin, respectively, and grown for further 48 h (since 48 h induction time was optimal in the experiment mentioned above). After centrifugation at 9000 rpm for 20 min, the supernatant from various time intervals was obtained, and then purified to be analyzed by SDS-PAGE (Mu et al., 2018).

### Protein Purification and Activation

The fermented culture from each sample was centrifuged at 9000 rpm for 20 min to collect 10 mL supernatant and purified by immobilized metal affinity chromatography (IMAC). A nickel-nitrilotriacetic acid (Ni-NTA) column (BBI Life Sciences) was used to combine the protein with a histidine-tag. The column resin was equilibrated twice with lysis buffer (300 mM NaCl, 50 mM NaH_2_PO_4_, 10 mM imidazole, pH 8.0), and then 10 mL supernatants were used to bind to the balanced column resin on a room rotor for 2 h. After washing twice with wash buffer (300 mM NaCl, 50 mM NaH_2_PO_4_, 20 mM imidazole, pH 8.0), the combined proteins were collected using one column volume of elution buffer (300 mM NaCl, 50 mM NaH_2_PO_4_, 250 mM imidazole, pH 8.0) and analyzed by reducing SDS-PAGE with Coomassie blue staining (Mu et al., 2018).

A final concentration of 200 μg/mL trypsin was used to digest 10 mL supernatants which were collected from either 12 h cultivation of *L. lactis* NZ9000 harboring pNZ8048-P*_p5_*-*SP_usp45_(K2A)-promtg* or 48 h cultivation of 5 ng/mL nisin-induced *L. lactis* NZ9000 harboring pNZ8048-*SP_usp45_*-*promtg*, respectively, the reaction was conducted at 37°C for 1 h. IMAC was used to purify mature MTG-6His (see above), analyzed by reducing SDS-PAGE with Coomassie blue staining (Mu et al., 2018) and stored for further use.

### Preparation of Magnetic Porous Fe_3_O_4_ Nanoparticles and Immobilization of MTG-6His

Magnetic porous Fe_3_O_4_ nanoparticles were produced according to the previous publication (Sheng et al., 2018) by adding FeCl_3_⋅6H_2_O (4 mmol) and NaAc⋅3H_2_O (12 mmol) into 60 ml EG with stirring for 1 h. The generated solution was heated at 180°C for 12 h. After cooling down to 25°C, the Fe_3_O_4_ nanoparticles were collected with magnet and observed with transmission electron microscope (TEM). Immobilized MTG was obtained by mixing mature MTG-6His with Fe_3_O_4_ nanoparticles dispersed in 0.1 M NHS-EDC solution. The parameters of MTG-6His/Fe_3_O_4_ ratio, pH and treatment time were investigated to generate immobilized enzyme, which was subsequently separated with external magnet, washed with water for three times and dried at 50°C. Scanning electron microscope was used for the observation of microstructure of nanoparticles. Immobilization rate was measured as follows:

$$Y=\frac{\mathrm{C0}-C\,1}{\mathrm{C0}}$$

where, C_0_ and C_1_ are the total MTG content before and after immobilization, respectively.

### Measurement of Enzyme Activity

Mature MTG-6His concentration was measured as previously described (Bradford, 1976) with a Bradford Protein Assay Kit (Thermo Fischer). Then a colorimetric hydroxamate procedure was carried out to test the enzymatic activity according to the literature (Grossowicz et al., 1950). Typically, MTG was mixed with the substrate solution, containing a final concentration of 100 mM hydroxylamine, 30 mM Z-Gln-Gly, 200 mM Tris/HCl-buffer, 10 mM reduced glutathione (pH 6.0). After 10 min of initial reaction at 50°C, the reaction was stopped by adding 160 μl terminal reagent containing 12% trichloroacetic acid, 3 M HCl, 5% FeCl_3_.6H_2_O (dissolved in 0.1 M HCl) at a volume ratio of 1:1:1. A spectrophotometer was used to measure the extinction of the reaction system at 525 nm. The definition of one unit of MTG-6His was the amount of mature MTG-6His needed for the formation of 1 μmol L-glutamic acid γ-monohydroxamate per minute at 50°C (pH 6.0).

### Crosslinking of Soy Protein Isolate by MTG-6His

Soy protein isolate (SPI) was mixed with distilled water in a ratio of 1:100 (w/v), and stirred to get full hydration at room temperature for 12 h. The SPI solution was centrifuged at 16,500 rpm for 10 min to reserve the supernatant containing soluble proteins. MTG-6His was then mixed with the SPI supernatant at a ratio of 1:1000 (w/v) and incubated at 50°C in a constant shaker at 200 rpm. Reacted samples were detected from various reaction time intervals at 15, 30, 60, and 120 min. SPI mixed with pro-MTG-6His/water/immobilized MTG were incubated for 120 min in the same conditions and used as two controls. Immobilized MTG and commercial *S. mobaraensis* MTG (Jiangsu Yiming Biological Co., Ltd., China) was used as a positive control. Finally, all the reacted samples and controls were applied to SDS-PAGE gels.

### The Protein Band Analysis

BANDSCAN software (Glyko Co., Ltd., United States) was used to analyze the protein bands in a semiquantitative level. Area and lanes on the SDS-PAGE electropherogram which needed to be analyzed was selected and numbered. The band with the highest intensive gray was set to be 100%. Bands in other lanes will generate their respective percentages of proteins based on their own gray intensity.

### Statistical Analysis

All tests were repeated in triplicate, and all values are shown as mean ± standard deviation (SD). All statistical analyses were committed with the software SPSS 13.0 (SPSS Inc., Chicago, IL, United States).

## Results

### Design of Soluble MTG Variants for Heterologous Expression

In order to obtain a soluble MTG that can be easily purified from culture supernatants we designed a gene encoding SP*_usp45_* fused to pro-MTG that contained a 6-histidine tag at the C-terminus (Figure 2). Unfortunately, the presence of a *Nco*I restriction site in the pro-region of MTG prevented a direct cloning and it became necessary to replace this site by *Bam*HI. This enabled efficient cloning in pNZ8048 under the control of P*_nisA_* (pNZ8048-*SP_usp45_(K2A)-promtg)*. The mutation (K2A) was eliminated by round PCR 2 rendering the plasmid pNZ8048-*SP_usp45_-promtg* (Figure 2). Next to the expression of engineered MTG under the control of nisin induction, we engineered a second variant in which the expression was constitutively driven from the P*_p5_* promoter in the vector pNZ8048-P*_p5_*-*SP_usp45_(K2A)-promtg* (Figure 2).

### SP*_usp45_* (K2A)/SP*_usp45_* Can Translocate Pro-MTG-6His

**FIGURE 3:**
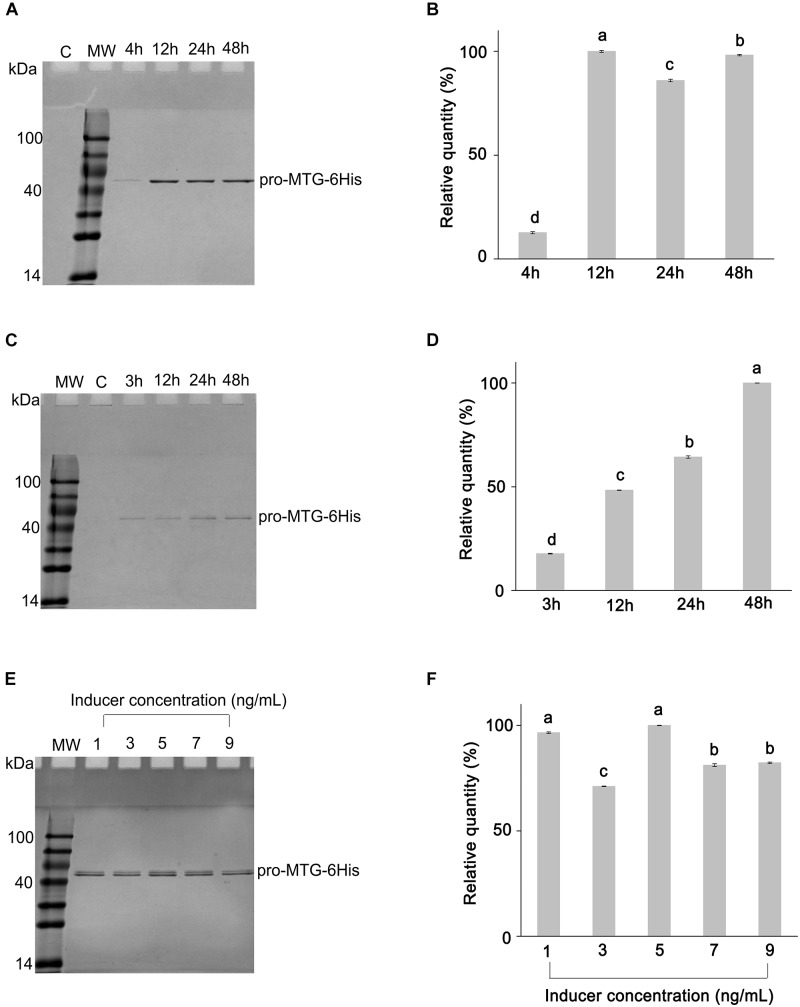
SDS-PAGE protein gel analysis of SP-MTG-6His produced by *L. lactis* strains. MW, molecular weight. **(A)** Purified pro-MTG-6His from *L. lactis* (pNZ8048-P*_p5_*-*SP_usp45_(K2A)-promtg*) with fermentation time of 4, 12, 24, and 48 h; Product from *L. lactis* (pNZ8048-P*_p5_*) with fermentation time of 48 h was treated as control (C). **(B)** The estimated relative quantities of pro-MTG-6His from *L. lactis* strains (pNZ8048-P*_p5_*-*SP_usp45_(K2A)-promtg*) with different fermentation time. **(C)** Purified pro-MTG-6His from 1 ng/mL nisin-induced culture of *L. lactis* (pNZ8048-*SP_usp45_*-*promtg*) with different induction times (3, 12, 24, and 48 h). Product from *L. lactis* (pNZ8048) with fermentation time of 48 h was treated as a control (C). **(D)** The estimated relative quantities of pro-MTG-6His from 1 ng/mL nisin-induced culture of *L. lactis* strains (pNZ8048-*SP_usp45_*-*promtg*) with different induction times (3, 12, 24, and 48 h). **(E)** Purified pro-MTG-6His from *L. lactis* strains (pNZ8048-*SP_usp45_*-*promtg*) with 48 h fermentation and different concentrations of nisin as inducer. **(F)** The estimated relative quantities of pro-MTG-6His from the culture of *L. lactis* strains (pNZ8048-*SP_usp45_*-*promtg*) with 48 h cultivation and different concentrations of nisin as inducer. Data with different letters above the error bars are significantly different at *P* < 0.01.

The vector pNZ8048-P*_p5_*-*SP_usp45_(K2A)-promtg* (Figure 2) was transformed into *L. lactis* NZ9000, obtaining *L. lactis* NZ9000 (pNZ8048-P*_p5_*-*SP_usp45_(K2A)-promtg*). After 4, 12, 24, and 48 h fermentation, pro-MTG-6His purified from *L. lactis* NZ9000 (pNZ8048-P*_p5_*-*SP_usp45_(K2A)-promtg*) was analyzed by SDS-PAGE and BANDSCAN. *L. lactis* NZ9000 (pNZ8048-P*_p5_*) was treated in the same way and used as a control. An approximately 43 kDa protein band corresponding to pro-MTG-6His was shown in four lanes except in the one with the negative control, indicating pro-MTG-6His was successfully secreted in *L. lactis* NZ9000 by promoter P*_p5_* (Figure 3A). The 12 h fermented sample produced the largest quantity of pro-MTG-6His compared to 4, 24, and 48 h fermented samples (7.9/1.2/1.1 times of the amount of 4 h/24 h/48 h production, respectively) (*P* < 0.05) (Figure 3B). The vector pNZ8048-*SP_usp45_*-*promtg* (Figure 2) was transformed into *L. lactis* NZ9000, obtaining *L. lactis* NZ9000 (pNZ8048-*SP_usp45_*-*promtg*). Supernatants taken from 1 ng/mL of nisin-induced culture of *L. lactis* NZ9000 (pNZ8048-*SP_usp45_*-*promtg*) with four different induction times (3, 12, 24, and 48 h) were purified, and 48 h induction of *L. lactis* NZ9000 (pNZ8048) was treated as control. An approximately 43 kDa protein band corresponding to pro-MTG-6His was shown in four lanes except in the one with the negative control, indicating pro-MTG-6His was successfully secreted by *L. lactis* by promoter P*_nisA_* (Figure 3C). A quantitative analysis (Figure 3D) showed that the secretion and recovery of soluble pro-MTG-6His increased with time (from 3 h to 48 h), being the sample induced for 48 h the most efficient (8.9/2.6/1.8 times of that of 3 h/12 h/24 h production) (*p* < 0.05). Once the optimal induction time was determined, we adjusted the concentration of nisin used to induce MTG expression in *L. lactis* NZ9000 (pNZ8048-*SP_usp45_*-*promtg*). At 5 ng/mL, we achieved the highest quantity of pro-MTG-6His (1.0, 1.4, 1.2 and 1.2 times of that of 1, 3, 7 and 9 ng/mL nisin, respectively) (*p* < 0.05) with fermentation time of 48 h (Figure 3E,F).

### Secretion of Pro-MTG-6His Has Slight Impact on the Growth of *L. lactis*

**FIGURE 4:**
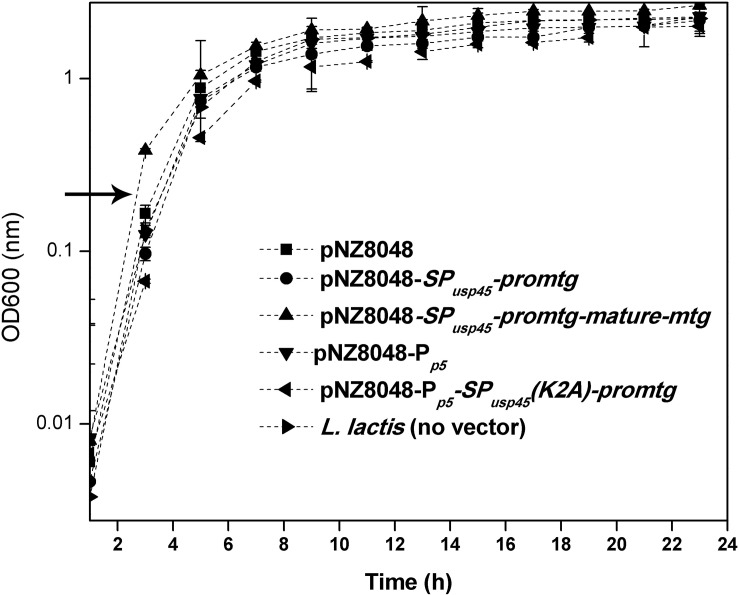
Growth curves of different *L. lactis* strains at 30°C. The arrow points the inducing time point. These values represent the means from three independent measurements.

To detect the impact that the secretion of pro-MTG-6His played on the growth of *L. lactis*, growth curves of *L. lactis* NZ9000 strains harboring four different vectors (pNZ8048-P*_p5_*, pNZ8048-P*_p5_*-*SP_usp45_(K2A)-promtg*, pNZ8048 and pNZ8048-*SP_usp45_*-*promtg*) or no vector were studied. As shown in Figure 4, growth profiles of *L. lactis* NZ9000 (pNZ8048-P*_p5_*) and *L. lactis* NZ9000 (pNZ8048) were almost coincident with that of *L. lactis* NZ9000 indicating that neither pNZ8048-P*_p5_* nor pNZ8048 affect the growth of *L. lactis* NZ9000. Unlike strains harboring other vectors, *L. lactis* NZ9000 (pNZ8048-P*_p5_*-*SP_usp45_(K2A)-promtg*) had a relative slower growth rate during the exponential phase. The OD_600_ of all *L. lactis* strains were stabilized around 2.8 after reaching stationary phase (Figure 4). In all cases, growth followed a similar trend so we conclude that the overexpression of MTG does not induce a significant toxicity in *L. lactis*.

### *L. lactis* Produces Functional MTG

**FIGURE 5:**
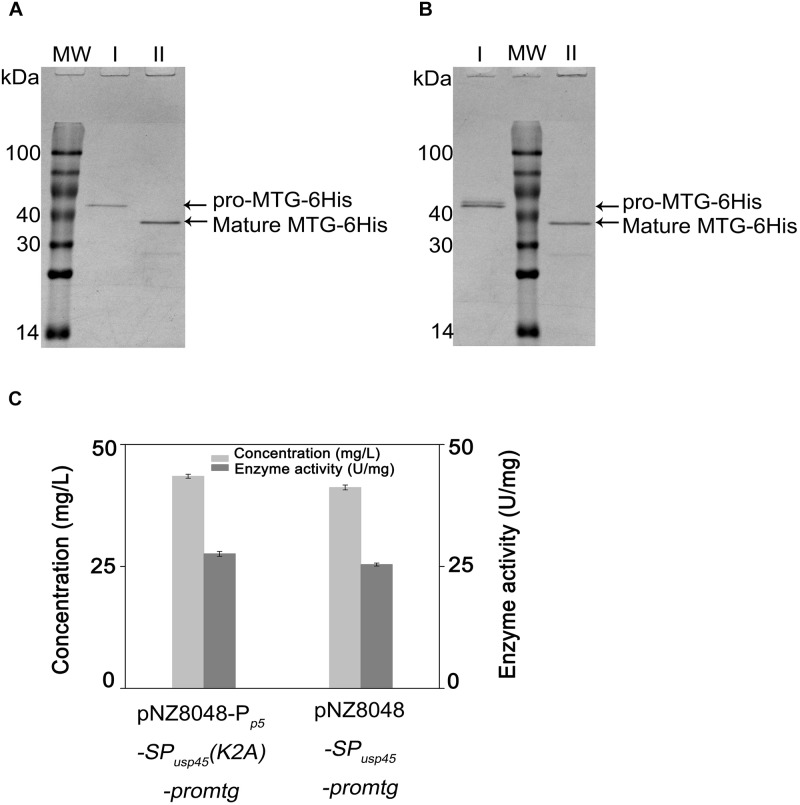
Analysis of trypsin activated pro-MTG-6His. MW: molecular weight. **(A)** Samples from *L. lactis* strains (pNZ8048-P*_p5_*-*SP_usp45_(K2A)-promtg*). **(B)** Samples from *L. lactis* strains (pNZ8048-*SP_usp45_*-*promtg*). **(C)** Enzymatic activity and concentration of MTG-6His produced by *L. lactis*. I. protein before trypsin digestion, II. protein activated with a final concentration of 200 μg/ml trypsin.

Pro-MTG-6His produced by *L. lactis* NZ9000 (pNZ8048-*SP_usp45_*-*promtg*) and *L. lactis* NZ9000 (pNZ8048-P*_p5_*-*SP_usp45_(K2A)-promtg*) under control of P*_nisA_* and P*_p5_*, respectively, were purified and digested by 200 μg/mL trypsin. Figure 5 shows that there is a single band with a molecular mass (38.9 kDa) corresponding to MTG-6His existing in the trypsin-treated samples (lane II), indicating that pro-MTG-6His produced by *L. lactis* under the control of either P*_nisA_* or P*_p5_* were completely activated by trypsin. The fully digested MTG-6His were tested to have a concentration of 70.5 ± 0.4 mg/L under the control of P*_p5_* and 65.2 ± 0.5 mg/L under the control of P*_nisA_* (Figure 5C).

MTG-6His activities were measured by a colorimetric hydroxamate procedure with Z-Gln-Gly to be a substrate. After full digestion, MTG-6His activity was 27.6 ± 0.5 U/mg when the expression was controlled by the promoter P*_p5_* (mutant K2A) and 25.4 ± 0.3 U/mg when the promoter P*_nisA_* controlled the expression (wild-type MTG sequence). The enzyme activities of MTG-6His measured above were in the range to what has been reported in previous studies (Salis et al., 2015).

### Preparations of Magnetic Porous Fe_3_O_4_ Nanoparticles and Immobilized MTG-6His

**FIGURE 6:**
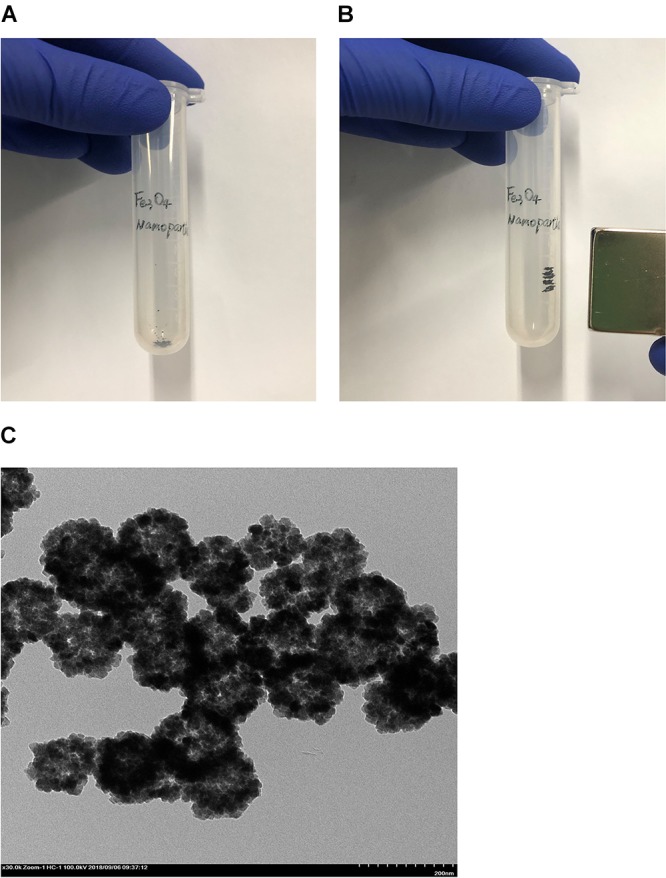
Verification of the magnetic Fe_3_O_4_. **(A)** The Fe_3_O_4_ particles not magnetized. **(B)** The particles can be attracted by a magnet Fe_3_O_4_. **(C)** Observation the structure of generated Fe_3_O_4_ nanoparticles by TEM.

The generated particles were easily attracted by a magnet indicating magnetic Fe_3_O_4_ has been generated successfully (Figure 6A,B). TEM was used to investigate the structure of generated Fe_3_O_4_ nanoparticles. As shown in Figure 6C. The surface of all particles was rough, convex and porous and the particle size ranged from 50 to 100 nm.

**FIGURE 7:**
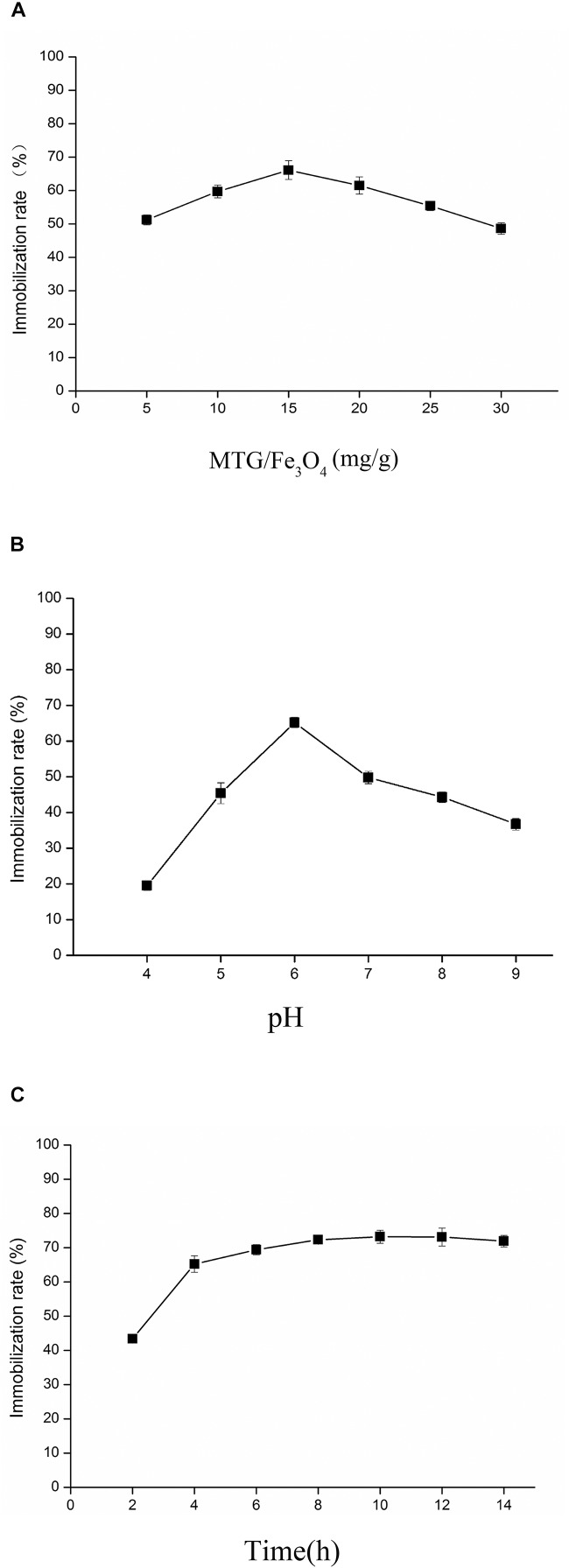
Analysis the influencing factors of immobilized enzymes from three aspects. **(A)** The effects of MTG-6His/Fe_3_O_4_ ratio. In the case of the same immobilizing time, when MTG-6His/Fe_3_O_4_ ratio was 15 mg/g, the immobilization rate was the highest. **(B)** The effects of pH. As MTG-6His/Fe_3_O_4_ ratio was 15 mg/g, the best pH for immobilizing is 6.0. **(C)** The effects of treatment time on immobilized MTG. When MTG-6His/Fe_3_O_4_ ratio was 15 mg/g, the pH was 6.0, the highest immobilization rate appeared in the 10 h.

The effects of MTG-6His/Fe_3_O_4_ ratio, pH and treatment time on immobilized MTG-6His were investigated. As shown in Figure 7A, after immobilizing for 6 h, when MTG-6His/Fe_3_O_4_ ratio was 15 mg/g, the immobilization rate of 65% was detected to be higher than that at other concentration ratios. With this MTG-6His/Fe_3_O_4_ ratio, the optimized pH 6.0 was obtained as indicated in Figure 7B where the highest immobilization rate was 67%. Additionally, the highest immobilization rate reached 73% when the immobilization time was set at 10 h (Figure 7C).

### Characterizations of Immobilized MTG-6His

**FIGURE 8:**
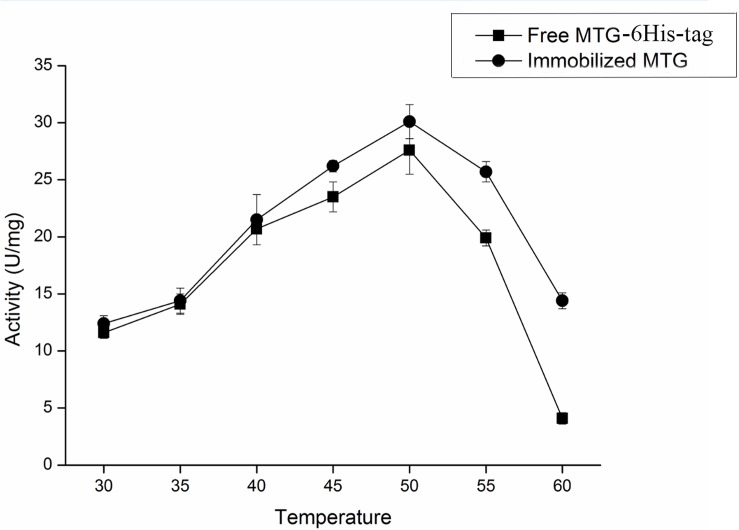
Effect of temperature on the activity of free and immobilized MTG enzyme. When the temperature reached 50°C, both of free and immobilized MTG enzyme have the highest activity. And the activity of immobilized MTG enzyme is higher than free.

**FIGURE 9:**
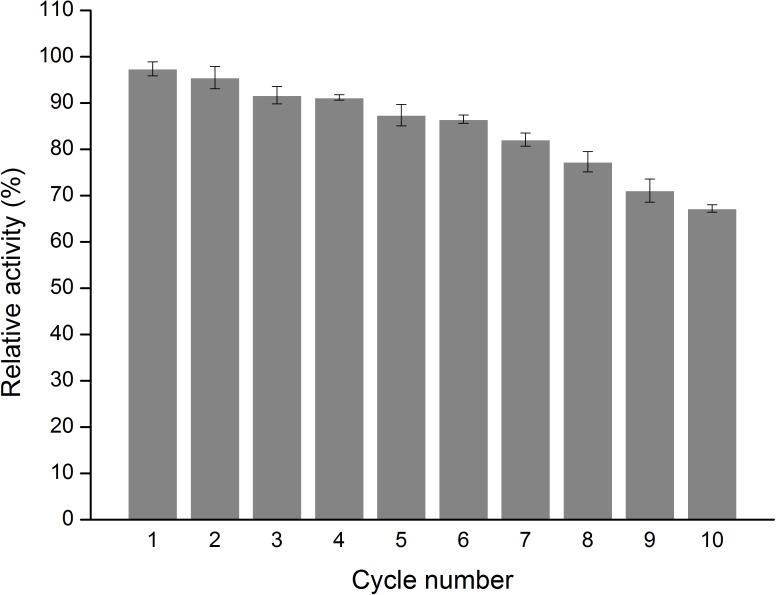
Measure the enzymatic activity of immobilized MTG. Determination of the reusability of immobilized enzymes. With the number of uses increases, the relative activity is decreasing, but the relative activity of the enzyme is still greater than 50% and reached 67%.

The effect of temperature on the activity of free and immobilized MTG-6His has been investigated. As shown in Figure 8 in the temperature range of 30–60°C the immobilization improved the activity up to 29.1 ± 0.4 U/mg although the optimum temperature remained 50°C. Reusability was detected by recycling the immobilized MTG-6His for 10 times and measuring its enzymatic activity at each time. As shown in Figure 9. The immobilized MTG-6His had good reusability with recovering 67% of its initial activity after 10 reuses.

### Results of SPI-Crosslinking by Free/Immobilized MTG-6His

**FIGURE 10:**
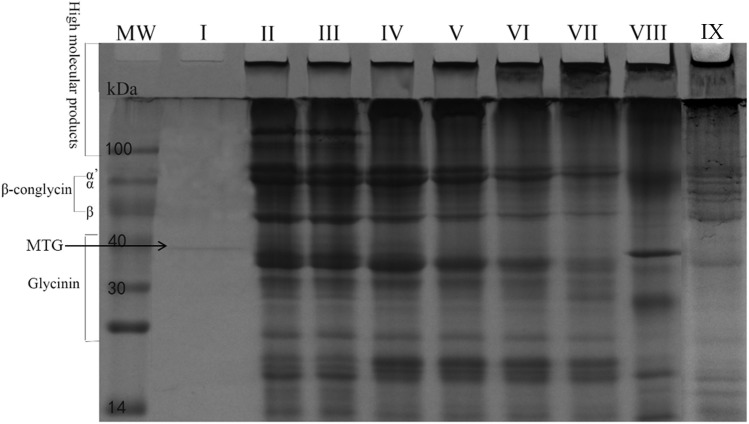
SPI crosslinking by MTG. Purified Mature MTG-6His from *L. lactis* strains. MW, molecular weight. I. Mature MTG-6His in water, II. SPI in water, III. SPI with pro-MTG-6His at 120 min, IV. SPI with mature MTG-6His at 15 min, V. SPI with mature MTG-6His at 30 min, VI. SPI with mature MTG-6His at 60 min, VII. SPI with mature MTG-6His at 120 min, VIII. SPI with commercial MTG at 120 min, IX. SPI with immobilized mature MTG-6His at 120 min.

A SPI crosslinking reaction was performed to further determine the enzymatic activity of MTG-6His produced by *L. lactis* using a protein mixture as a substrate. In lanes where samples’ pro-regions were removed, aggregation of protein on the top of the separating gel and the stacking gel were observed while β-conglycinin and glycinin disappeared (Figure 10). When the pro-region was not removed from pro-MTG-6His, therefore no active MTG was present, SPI could not be cross-linked and no change was observed in the lane III proving that only mature MTG-6His could catalyze the crosslinking. Immobilized MTG-6His and free MTG-6His produced by *L. lactis* in this work can crosslink the SPI more intensively and produce higher molecular weight proteins compared to commercial MTG (lane VIII) which has been reacted with SPI in the same conditions (Lane IX).

## Discussion

Microbial transglutaminase is a biotechnologically relevant enzyme that meets applications in several fields. A broader use is hampered by the costs associated to its production and purification from the native producer organisms as well as heterologous host. As we show in this study, the cloning of *mtg* fused to SP*_usp45_*, under the control of the P*_nisA_* inducible promoter in the plasmid pNZ8048 or the P*_p5_* constitutive promoter in the plasmid pNZ8048-P*_p5_*, has permitted the production and secretion of pro-MTG-6His by *L. lactis*NZ9000, which is a widely used for industrial/medical protein production, such as alanine dehydrogenase and exendin-4 (Ye et al., 2010; Zeng et al., 2017).

In this study, Sec-dependent signal peptide SP*_usp45_* is shown to secrete MTG-6His up to 65.2 ± 0.5 mg/L while SP*_usp45_* (K2A) is shown to secret MTG-6His up to 70.5 ± 0.4 mg/L from *L. lactis*. This indicates that the replacement of lysine at position 2 by alanine of the signal peptide SP*_usp45_*, which was introduced due to the removal of a *Nco*I restriction site, does not have an impact on the secretion of heterologous MTG-6His in *L. lactis*. It has been advised that Sec translocation machinery would interact with the positively charged N-region of SP*_usp45_* (Tjalsma et al., 2000). Considering downstream position of the mutation K2A in SP*_usp45_* (K2A), this might not affect the overall positive charge necessary for the correct interaction with the Sec translocation machinery.

Compared to the inducible expression from the promoter P*_nisA_*, the constitutive expression with promoter P*_p5_* achieved higher production of MTG-6His, indicating that the constitutive expression can be more favorable than the nisin-inducible expression when prolonged fermentation times are required. Even more, as no inducer needs to be added and the production is optimal after 12 h, production costs will be reduced through the constitutive expression (Berenbaum and Zangerl, 1994; Zhu et al., 2017).

Production of pro-MTG-6His under the control of the constitutive promoter P*_p5_* slightly slowed the growth of the producer strain (Figure 4). The possible explanation might be much energy and substance that should be supplied for normal cell metabolism streams for the generation of pro-MTG-6His while in the case of system controlled by inducible promoter P*_nisA_*, this process was buffered by adding nisin into the culture in exponential growth phase to initiate expression. These results stand in line with previous studies where GFP was expressed under the Zinc-inducible promoter P*_czcD_* when *L. lactis* was used as the host strain (Mu et al., 2013).

Microbial transglutaminase-6His/Fe_3_O_4_ ratio is an important parameter affecting the immobilization rate. When the ratio is 15 mg/g, the rate reached 65%, higher than these at other ratios. In spite of high MTG-6His/Fe_3_O_4_ ratio increased binding chances of MTG-6His onto the nanoparticle surface, too much MTG-6His would block the binding between surface active sites and enzymes by their aggregation (Ling et al., 2016). Immobilized MTG-6His displayed higher activity than free MTG-6His at all tested temperatures. This might be explained by the fact that immobilization conferred enzyme with stronger conformation.

The crosslinking experiment on SPI has demonstrated the goodactivity of MTG-6His secreted by *L. lactis* for industrial application. Commercial *S. mobaraensis* MTG was added at high concentration producing an obvious band compared to the *L. lactis*-produced MTG-6His that was added at lower amount. Nevertheless, commercial MTG-crosslinking effect on SPI is relatively poorer than that of *L. lactis*-produced MTG-6His (Figure 10). One feasible reason is that commercial MTG was not completely purified since there were several unknown protein bands existing in lane VIII or it lost part of the activity during storage (Figure 10).

## Conclusion

In this study, Microbial transglutaminase (MTG) from *S. mobaraensis* has been secreted and purified from *L. lactis* under the control of both inducible and constitutive promoters. We provide an efficient way to produce high-quality MTG in a GRAS strain *L. lactis*. Magnetic immobilized MTG-6His prepared by porous Fe_3_O_4_ nanoparticles and MTG showed an higher activity of 29.1 ± 0.4 U/mg than free MTG-6His and retained 67% of its initial activity after 10 reuses. Our results provide a safe and easy-to-purify host strain and an easy-to-reuse magnetic immobilized MTG-6His for future MTG bioengineering work.

## Data Availability

The raw data supporting the conclusions of this manuscript will be made available by the authors, without undue reservation, to any qualified researcher.

## Author Contributions

DM designed this experiment and guided students’ work, and provided a lot of financial support for the research together with ZZ. JL constructed recombinant expression vectors, and was assisted by TM to complete the purification and activity determination of MTG-6His. TM and JL wrote the first draft of the manuscript and produced figures. JZ provided magnetic porous Fe_3_O_4_ nanoparticles and method for preparing nanoparticles. All authors of this manuscript have read and approved the final version submitted and no conflict of interest exists in this research. At the same time, the study design was approved by the appropriate biosafety review boards.

## Conflict of Interest Statement

The authors declare that the research was conducted in the absence of any commercial or financial relationships that could be construed as a potential conflict of interest.

## Abbreviations

Cm^R^: chloramphenicol resistance
GRAS: generally recognized as safe
OD: optical density
SDS: sodium dodecyl sulfate
TSBY medium: trypticase soy broth supplemented with 0.5% (v/v) yeast extract
Z-Gln-Gly: N-benzyloxycarbonyl-L-glutaminylglycine
